# Mortality and resource utilization in surgical versus transcatheter repeat mitral valve replacement: A national analysis

**DOI:** 10.1371/journal.pone.0301939

**Published:** 2024-05-23

**Authors:** Nguyen K. Le, Nikhil Chervu, Saad Mallick, Amulya Vadlakonda, Shineui Kim, Joanna Curry, Peyman Benharash

**Affiliations:** 1 Cardiovascular Outcomes Research Laboratories (CORELAB), David Geffen School of Medicine, University of California, Los Angeles, Los Angeles, CA, United States of America; 2 David Geffen School of Medicine, University of California, Los Angeles, UCLA, Los Angeles, CA, United States of America; 3 Department of Surgery, David Geffen School of Medicine, University of California, Los Angeles, UCLA, Los Angeles, CA, United States of America; Bern University Hospital, SWITZERLAND

## Abstract

**Background:**

Transcatheter mitral valve replacement (TMVR) has garnered interest as a viable alternative to the traditional surgical mitral valve replacement (SMVR) for high-risk patients requiring redo operations. This study aims to evaluate the association of TMVR with selected clinical and financial outcomes.

**Methods:**

Adults undergoing isolated redo mitral valve replacement were identified in the 2016–2020 Nationwide Readmissions Database and categorized into TMVR or SMVR cohorts. Various regression models were developed to assess the association between TMVR and in-hospital mortality, as well as additional secondary outcomes. Transseptal and transapical catheter-based approaches were also compared in relation to study endpoints.

**Results:**

Of an estimated 7,725 patients, 2,941 (38.1%) underwent TMVR. During the study period, the proportion of TMVR for redo operations increased from 17.8% to 46.7% (nptrend<0.001). Following adjustment, TMVR was associated with similar odds of in-hospital mortality (AOR 0.82, p = 0.48), but lower odds of stroke (AOR 0.44, p = 0.001), prolonged ventilation (AOR 0.43, p<0.001), acute kidney injury (AOR 0.61, p<0.001), and reoperation (AOR 0.29, p = 0.02). TMVR was additionally correlated with shorter postoperative length of stay (pLOS; β -0.98, p<0.001) and reduced costs (β -$10,100, p = 0.002). Additional analysis demonstrated that the transseptal approach had lower adjusted mortality (AOR 0.44, p = 0.02), shorter adjusted pLOS (β -0.43, p<0.001), but higher overall costs (β $5,200, p = 0.04), compared to transapical.

**Conclusions:**

In this retrospective cohort study, we noted TMVR to yield similar odds of in-hospital mortality as SMVR, but fewer complications and reduced healthcare expenditures. Moreover, transseptal approaches were associated with lower adjusted mortality, shorter pLOS, but higher cost, relative to the transapical. Our findings suggest that TMVR represent a cost-effective and safe treatment modality for patients requiring redo mitral valve procedures. Nevertheless, future studies examining long-term outcomes associated with SMVR and TMVR in redo mitral valve operations, are needed.

## Introduction

Mitral regurgitation is the most common valvular disorder in the United States, affecting more than two million individuals [1]. In recent times, an increasing proportion of patients choose a bioprosthetic valve to avoid the need for lifelong anticoagulation [2, 3]. Importantly, several investigators have reported a concomitant rise in the need for reoperation attributable to hastened degeneration of biological valves compared to mechanical prostheses [4–7].Thus, surgeons and cardiologists are faced with decisions regarding the optimal management of patients who require multiple valve replacements over their lifetime.

Given the significant morbidity and mortality associated with repeat sternotomy, transcatheter mitral valve replacement (TMVR) has garnered attention as a less invasive alternative to the traditional surgical mitral valve replacement (SMVR) [8, 9]. Nevertheless, the use of TMVR remains controversial due to the complex anatomy of the mitral valve, technical challenges of the transcatheter approach, and lack of prospective or large-scale analyses [10–12]. The available literature comparing the two approaches have demonstrated TMVR to have lower or equivalent mortality, shorter length of stay, and lower hospitalization costs, relative to SMVR [13, 14]. However, these studies comprised of fewer than 400 estimated patients in each treatment group and may not be able to change practice [13, 14].

In the present work, we examined the clinical and financial outcomes of TMVR and repeat SMVR using a large nationally representative cohort. We hypothesized TMVR to be associated with lower mortality, complication rates, hospitalization costs, and shorter postoperative length of stay, compared to SMVR.

## Methods

This was a retrospective study of the 2016 to 2020 Nationwide Readmissions Database (NRD). Maintained by the Healthcare Cost and Utilization Project (HCUP), the NRD is the largest all-payer readmission database in the US [15]. It contains discharge data from 31 states in the country and utilizes center-specific discharge weights to provide accurate estimates for approximately 60% of all U.S hospitalizations. Additionally, each patient is assigned a unique linkage number, allowing for tracking of readmissions across hospitals within each calendar year.

**Fig 1:**
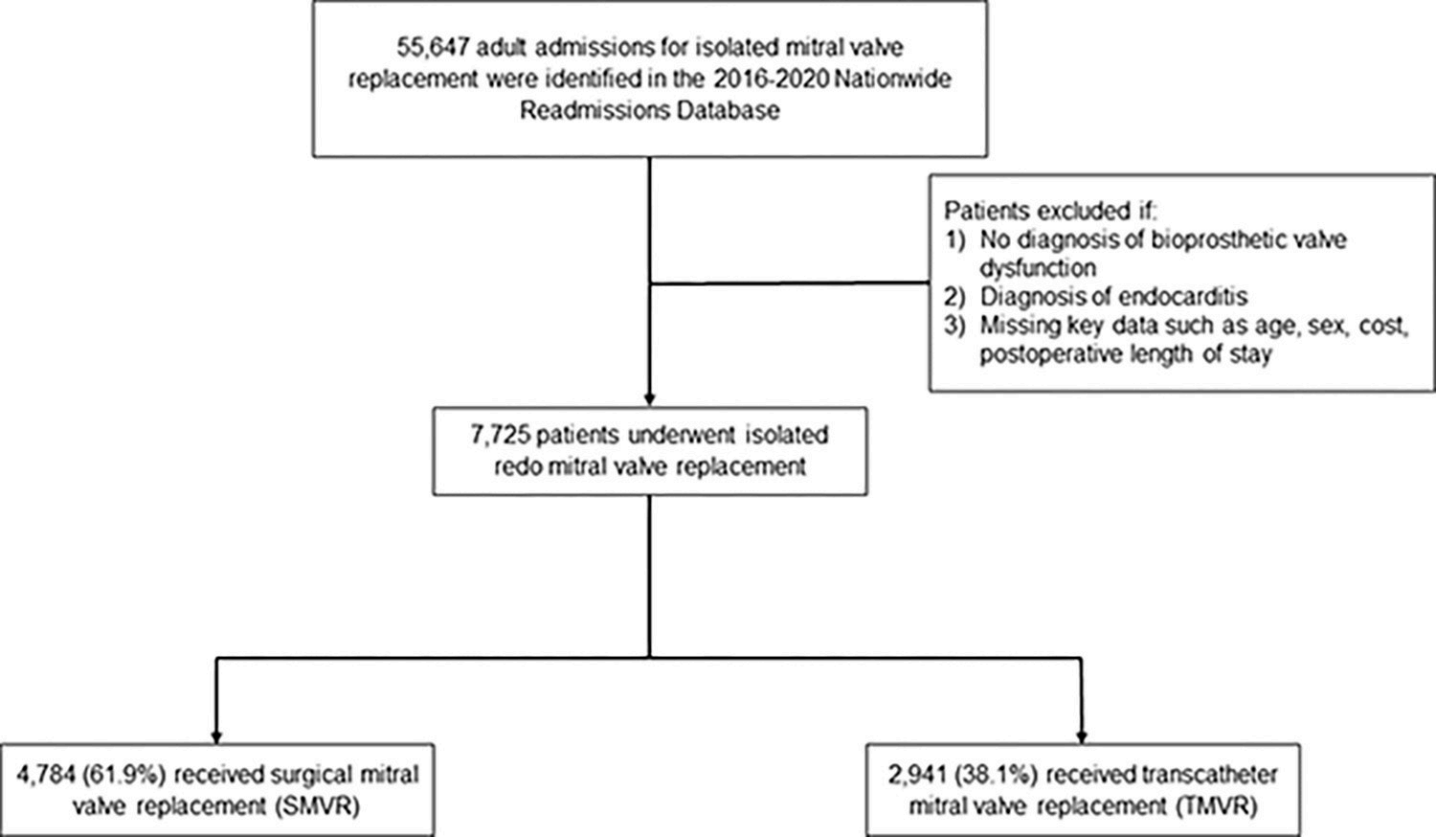
Study flowchart of survey-weighted estimates. Of the 55,647 adult hospitalizations for isolated mitral valve replacements identified in the 2016–2020 NRD, 7,725 patients were included in this study. Of these, 2,941 (38.1%) underwent transcatheter mitral valve replacement. All estimates represent survey-weighted methodology.

Using relevant *International Classification of Disease*, *10*^*th*^ *Revision* (ICD-10) codes, we identified all adults (≥ 18 years) with a diagnosis of bioprosthetic valve dysfunction who subsequently underwent TMVR or SMVR (S1 Table). Based on prior literature, this cohort was defined to have received a repeat valve procedure [13, 14]. Furthermore, those undergoing both TMVR and SMVR on the same index operation were classified as SMVR, as they ultimately received a more invasive intervention. Those undergoing concomitant cardiac procedures, including coronary artery bypass grafting, aortic valve replacement or repair, pulmonic valve replacement or repair, tricuspid valve replacement or repair, or mitral valve repair, were excluded. Patients with history of endocarditis or missing data for age, sex, hospitalization charges, or postoperative length of stay (pLOS), were excluded from analysis to reduce heterogeneity (1.1%; Fig 1).

Hospital and patient characteristics were defined according the NRD data dictionary [15]. Patient comorbidities and complications were ascertained using relevant ICD-10 codes (S1 Table). The Van Walraven modification of the Elixhauser Comorbidity Index was used to quantify each patient’s burden of chronic conditions [16, 17]. Costs were calculated from hospitalization charges by applying center-specific cost-to-charge ratios and adjusted for inflation using the 2020 Personal Health Index [18, 19]. Annual institutional volumes of SMVR and TMVR, inclusive of initial and repeat interventions, were tabulated and examined in a continuous manner. To calculate pLOS, the day that TMVR or SMVR was performed was first ascertained using the *prday* variable from the NRD data dictionary. pLOS was subsequently calculated as the difference between the total length of stay (NRD variable *los*) and the day of operation.

The primary outcome of interest was in-hospital mortality. Secondary endpoints included perioperative complications, pLOS, hospitalization costs, non-home discharge, and 30-day non-elective readmissions. Complications of interest were derived from Society of Thoracic Surgeons Performance Measures. These included stroke or transient ischemic attack (TIA), prolonged ventilation (>96 hours), acute kidney injury (AKI), reoperation, major bleeding, and vascular complications [20]. Major bleeding consisted of gastrointestinal bleed, postoperative hematoma or hemorrhage, hemoptysis, epistaxis, intracranial hemorrhage, and requirement for blood transfusion [21, 22]. Meanwhile, vascular complications comprised of injuries to blood vessels, accidental puncture or laceration of a circulatory system organ or structure, and acute limb ischemia [23–25]. Non-home discharge was defined as discharge to an acute care hospital, intermediate care facility, or a skilled nursing facility.

Cuzick’s nonparametric test (nptrend) was used to assess the significance of temporal trends [26]. The Mann-Whitney U test and Pearson’s *χ*^2^ tests were utilized to examine the significance of intergroup differences for continuous and categorical variables, respectively. Covariate selection was guided using the Least Absolute Shrinkage Selection Operator (LASSO), a regularization method that enhances model generalizability by reducing collinearity and overfitting [27]. LASSO was performed with 10-fold cross-validation, and λ = 0.0068966 was chosen to minimize the Bayesian information criterion (BIC). Following LASSO, covariates selected to be incorporated into the entropy balancing model included institutional annual SMVR and TMVR volumes, age, sex, elective status, Elixhauser Index, and comorbidities such as liver disease, neurologic conditions, coagulopathy, and chronic lung disease. Entropy balancing was subsequently used to adjust for differences in patient and hospital characteristics between *TMVR* and *SMVR*. Unlike propensity matching, entropy balancing assigns sample weights to balance distribution of covariates. Thus, it does not rely on specific propensity score models and retains the entire sample for analysis [28–30]. The product of entropy balancing weight and center-specific discharge weight was subsequently computed and assigned to each patient. Linear, Poisson, and logistic regression models were developed to determine the association of TMVR or SMVR and outcomes of interest, as appropriate.

A subgroup analysis was similarly conducted to compare the differences in clinical and financial endpoints between the transapical (TA-TMVR) and transseptal (TS-TMVR) approaches of TMVR. Given the similar patient characteristics between *TA-TMVR* and *TS-TMVR* (S2 Table), no entropy balancing was utilized. Linear, Poisson, and logistic regression models were constructed to evaluate the relationship between type of transcatheter access and endpoints of interest.

The number of patients reported in this study are survey-weighted estimates as reported by HCUP. Continuous variables are reported as medians with interquartile ranges (IQR), and categorical variables are presented as percentages (%). Regression outputs are reported as adjusted odds ratios (AOR) or as beta coefficients (β) with 95% confidence intervals (CI), as appropriate. Statistical significance was set at α = 0.05. All statistical analyses were performed using Stata 16.0 (StataCorp, College Station, TX). The Stata codes used to conduct the analysis are also reported in the S4 Table. Given the de-identified nature of the NRD, the study was deemed exempt from full review by the Institutional Review Board at the University of California, Los Angeles.

## Results

### Cohort characteristics

**Fig 2:**
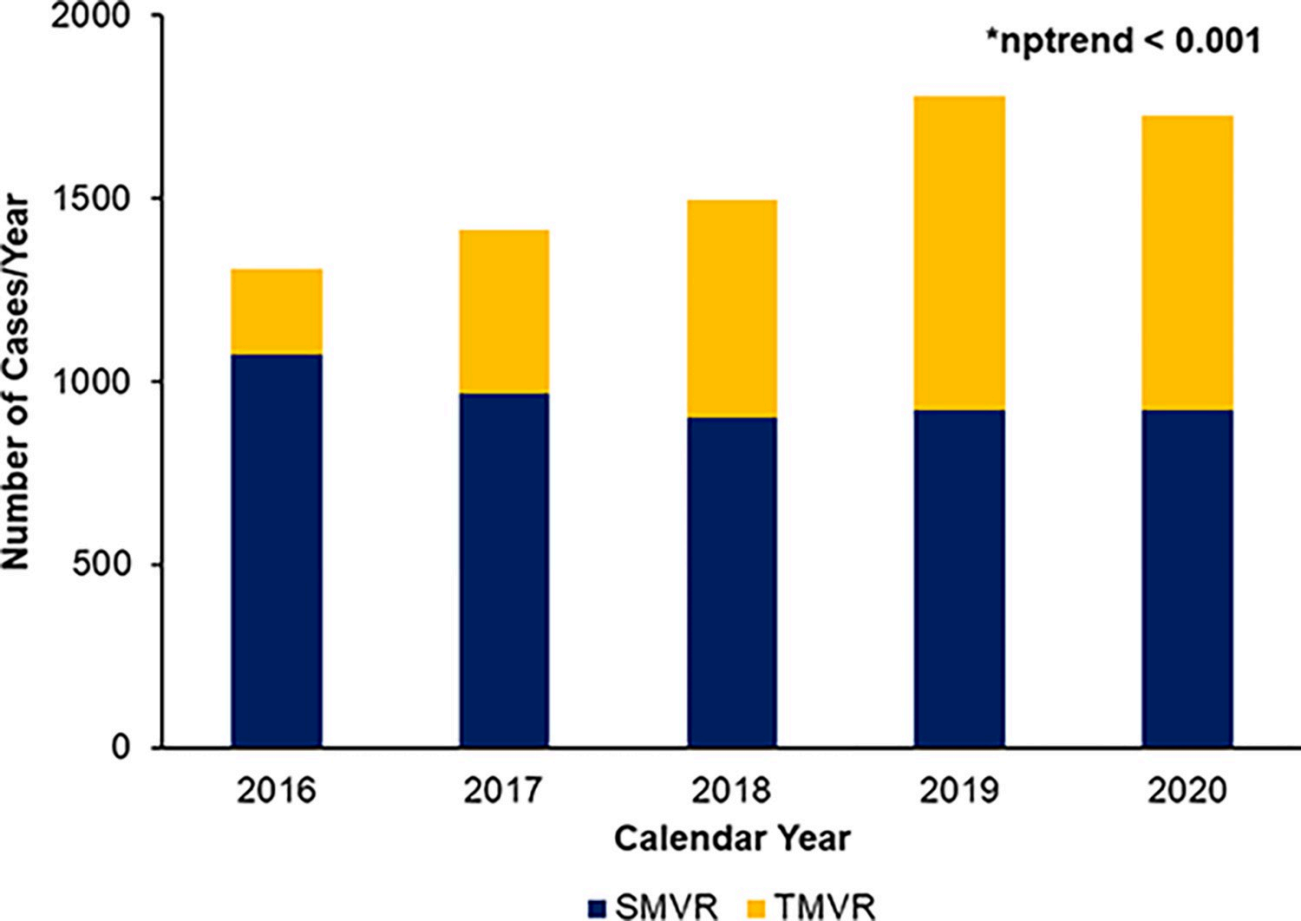
Annual trends in the volumes of surgical mitral valve replacement (SMVR) and transcatheter mitral valve replacement (TMVR), 2016–2020. The proportion of TMVR procedures significantly increased over the study period, nptrend < 0.001.

Of 7,725 patients meeting inclusion criteria, 2,941 (38.1%) received TMVR. The proportion of patients receiving TMVR increased from 17.8% in 2016 to 46.7% in 2020 (nptrend < 0.001; Fig 2). During the study period, the numbers of hospitals performing redo mitral valve operations increased from 296 to 306. Patient characteristics are detailed in Table 1. Compared to *SMVR*, *TMVR* were older (75 years [67–81] vs 65 [55–73], p < 0.001), but similar in the distribution of sex (female: 59.4 vs 57.5%, p = 0.23) and Elixhauser index (6 [5–8] vs 6 [5–8], p = 0.29). Although *TMVR* and *SMVR* had similar income distributions, TMVR patients were more commonly covered by Medicare (82.9 vs 56.9%, p < 0.001) and more likely to receive treatment at large (79.4 vs 73.9%, p = 0.002), metropolitan teaching (92.0 vs 86.1%, p = 0.001) institutions.

**Table 1:** Demographic, clinical, and hospital characteristics.

	*SMVR*	*TMVR*	p-value
	(n = 4,784)	(n = 2,941)	
Age (years, median, IQR)	65 [55–73]	75 [67–81]	<0.001
Female (%)	57.5	59.4	0.23
Elixhauser Index (median, IQR)	6 [5–8]	6 [5–8]	0.29
*Payer Status (%)*			<0.001
Private	27.1	9.6	
Medicare	56.9	82.9	
Medicaid	11.7	5.1	
Uninsured/Other	4.1	2.3	
*Income Quartile (%)*			0.09
76th - 100th	18.7	21.1	
51st - 75th	25.7	27.9	
26th - 50th	28.5	27.0	
0 - 25th	25.3	22.6	
*Hospital Status (%)*			0.001
Non-Metropolitan	1.9	1.0	
Non-Teaching Metropolitan	12.0	7.0	
Teaching Metropolitan	86.1	92.0	
*Hospital Size (%)*			0.002
Large	73.9	79.4	
Medium	19.3	17.1	
Small	6.7	3.5	
*Comorbidities (%)*			
Cardiac Arrhythmia	81.5	76.3	<0.001
Congestive Heart Failure	53.3	74.1	<0.001
Chronic Lung Disease	27.3	34.9	<0.001
Coagulopathy	39.7	19.4	<0.001
Diabetes	23.2	28.3	<0.001
End Stage Renal Disease	25.7	40.0	<0.001
Liver Disease	6.0	6.6	0.44
Other Neurologic Condition	13.3	7.0	<0.001
Pulmonary Circulatory Disease	43.6	52.3	<0.001

Reported as proportions unless otherwise noted. Statistical significance was set at p < 0.05.

*SMVR, surgical mitral valve replacement; TMVR, transcatheter mitral valve replacement; IQR, interquartile range.

### Unadjusted outcomes

On bivariate comparison, in-hospital mortality rates were similar in groups (TMVR: 4.4 vs SMVR: 4.8%, p = 0.58). Major complications were overall less frequent with the transcatheter approach, as evidenced by lower rates of stroke/TIA (1.7 vs 5.2%, p < 0.001), prolonged mechanical ventilation (2.9 vs 8.0%, p < 0.001), AKI (20.2 vs 29.5%, p < 0.001), reoperation (0.7 vs 2.3%, p = 0.003), and major bleeding (7.8 vs 9.9%, p = 0.03), while rates of vascular complications were similar (3.7 vs 3.4%, p = 0.64). The TMVR group also experienced shorter pLOS (2 days [1–5] vs 9 [6–15]), accrued lower hospitalization costs ($55,900 [42,200–79,500] vs 64,800 [46,700–95,000]), and faced lower rates of non-home discharge (16.6 vs 31.9%, all p < 0.001), compared to SMVR. At 30-days post-discharge, non-elective readmissions were similar between the two groups (15.4 vs 13.8%, p = 0.22) (Table 2).

**Table 2:** Unadjusted outcomes of patients undergoing surgical (SMVR) or transcatheter (TMVR) mitral valve replacement.

	*SMVR*	*TMVR*	p-value
	(n = 4,784)	(n = 2,941)	
In-Hospital Mortality (%)	4.8	4.4	0.58
*Major Complications (%)*			
Stroke/TIA	5.2	1.7	<0.001
Prolonged Ventilation	8.0	2.9	<0.001
Acute Kidney Injury	29.5	20.2	<0.001
Reoperation	2.3	0.7	0.003
Major Bleeding	9.9	7.8	0.03
Vascular Complications	3.4	3.7	0.64
*Resource Utilization*			
pLOS (days, median, IQR)	9 [6–15]	2 [1–5]	<0.001
Costs ($1,000s, median, IQR)	64.8 [46.7–95.0]	55.9 [42.2–79.5]	<0.001
Nonhome Discharge (%)	31.9	16.6	<0.001
30-Day Readmission (%)	13.8	15.4	0.22

Outcomes reported as proportions unless otherwise noted.

* TIA, transient ischemic attack; pLOS, postoperative length of stay; IQR, interquartile range

### Adjusted outcomes

A logistic model was developed to model in-hospital mortality and yielded adequate discrimination (C-statistic = 0.76). Following entropy balancing and risk adjustment, TMVR was found to be associated with similar odds of in-hospital mortality (AOR 0.82, 95%CI [0.48, 1.42]). TMVR was similarly linked to lower odds of stroke (AOR 0.44, 95%CI [0.27, 0.72]), prolonged ventilation (AOR 0.43, 95%CI [0.27, 0.67]), AKI (AOR 0.61, 95%CI [0.47, 0.79]), or reoperation (AOR 0.29, 95%CI [0.11, 0.79]), but similar odds of major bleeding (AOR 0.79, 95%CI [0.57, 1.11]) or vascular complications (AOR 1.56, 95%CI [0.89, 2.72]). Moreover, compared to SMVR, TMVR experienced shorter pLOS (β -0.98, 95%CI [-1.09, -0.86]) and decreased inpatient costs (β -$10,100, 95%CI [–16,500, –3,700]). Finally, the TMVR approach was associated with lower odds of non-home discharge (AOR 0.23, 95%CI [0.16, 0.31]), but similar odds of 30-day readmission (AOR 0.92, 95%CI [0.65, 1.29]) (Table 3).

**Table 3:** Adjusted outcomes of patients undergoing transcatheter (TMVR) mitral valve replacement, compared to surgical (SMVR) mitral valve replacement.

	AOR/β with 95% CI	p-value
In-Hospital Mortality (AOR)	0.82 [0.48, 1.42]	0.48
*Major Complications (AOR)*		
Stroke/TIA	0.44 [0.27, 0.72]	0.001
Prolonged Ventilation	0.43 [0.27, 0.67]	<0.001
Acute Kidney Injury	0.61 [0.47, 0.79]	<0.001
Reoperation	0.29 [0.11, 0.79]	0.02
Major Bleeding	0.79 [0.57, 1.11]	0.17
Vascular Complications	1.56 [0.89, 2.72]	0.12
*Resource Utilization*		
pLOS (β, days)	-0.98 [-1.09, -0.86]	<0.001
Costs (β, $1,000s)	-10.1 [-16.5, -3.7]	0.002
Nonhome Discharge (AOR)	0.23 [0.16, 0.31]	<0.001
30-Day Readmission (AOR)	0.92 [0.65, 1.29]	0.62

Outcomes reported as Adjusted Odds Ratio (AOR) or β Coefficient, with 95% confidence intervals (CI).

* TIA, transient ischemic attack; pLOS, postoperative length of stay

### Subgroup analysis

Among the transcatheter group across the study period, the proportion of trans-septal transcatheter mitral valve replacement (TS-TMVR) increased from 62.0% in 2016 to 91.3% in 2020 (nptrend < 0.001, S1 Fig). *TS-TMVR* and *TA-TMVR* cohorts were similar in terms of age, sex, burden of comorbidities, insurance coverage, and income distribution (S2 Table). Compared to *TA-TMVR*, *TS-TMVR* more frequently received care at large (80.1 vs 75.4%, p = 0.002) and metropolitan teaching (92.6 vs 88.7%, p = 0.03) hospitals. After adjustment, the transseptal approach had lower odds of in-hospital mortality (AOR 0.44, 95%CI [0.22, 0.87]), relative to the transapical approach. Nonetheless, TS-TMVR had similar odds of stroke/TIA, prolonged ventilation, AKI, reoperation, major bleeding, and vascular complications compared to TA-TMVR (S3 Table). With TA-TMVR as reference, TS-TMVR was associated with shorter pLOS (β -0.43, 95%CI [-0.59, -0.27]), but significantly increased adjusted costs (β +$5,200, 95%CI [+300, +10,200]). Finally, the transseptal approach was linked to similar odds of nonhome discharge (AOR 0.98, 95%CI [0.53, 1.80]) and 30-day readmission (AOR 1.20, 95%CI [0.79, 1.82]).

## Discussion

As the number of annual TMVR cases in redo operations continues to rise [31], large-scale examination of surgical and transcatheter approaches is necessary. We used a contemporary national representative cohort of repeat mitral valve replacement patients and made several important observations. We found the proportion of TMVR utilization to have more than doubled, now encompassing nearly half of all repeat mitral valve replacements. After adjusting for patient and hospital characteristics, TMVR was associated with similar odds of in-hospital mortality, fewer complications, shorter pLOS, and lower costs, compared to SMVR. Finally, we identified the transseptal approach to be linked to lower odds of in-hospital mortality, shorter LOS, and higher costs, compared to the transapical method. With implications for shared decision making, our findings merit further discussion.

The observed increase in transcatheter utilization for redo mitral valve replacement is consistent with prior reports [13, 14, 31, 32]. However, several concerns regarding the anatomical and technical challenges of transcatheter valve placement remain [10, 11]. One such area of discussion is the development of left ventricular outflow tract obstruction (LVOTO). LVOTO is a known rare complication with a reported incidence of 1–2% in SMVR and 5% in TMVR [32–35]. It is caused by the attachment of the anterior leaflet to the left ventricular outflow tract, thereby restricting flow [36]. The complication has been shown to be an independent predictor of mortality after TMVR, but prophylactic measures such as septal ablation may be utilized to help mitigate its incidence [37, 38]. Besides LVOTO, the large delivery system of TMVR also presents as a barrier that limits the adoption of this approach. Since the mitral valve is a bigger valve, it requires a larger prosthesis, transported via correspondingly larger sheaths [39]. Yet, this sizable set up imposes a challenge in maneuvering in a tight space and angling the prosthesis at the appropriate mitral annular plane [10]. To address this issue, newer TMVR technologies have been designed with smaller delivery apparatus while still accommodating for the large prostheses. The smallest apparatus being tested to date is measured at 20 French, close to the 14-French size observed in sheaths for transcatheter aortic valve replacement [40, 41]. With evolving technologies available, improving protocols, and increasing familiarity with the transcatheter approach, TMVR is gaining acceptance as an alternative for high-risk repeat procedures [31]. Thus, ongoing examination of the clinical and financial efficacy associated with TMVR will be necessary in the coming years.

Our study adds to mounting literature suggesting TMVR to be cost-effective with safe perioperative clinical outcomes, with an observed mortality rate of ~4.5% across both cohorts [6, 7, 32]. Similar to other national studies, we found TMVR to have equivalent adjusted odds of in-hospital mortality compared to SMVR, even after entropy balancing [13, 14]. We further identified TMVR to experience fewer postoperative complications than SMVR. This is congruent with prior literature, despite the frequent utilization of TMVR among patients with high surgical risk. In a study of 78 patients in Italy, Simonetto et al. found that TMVR patients have a lower rate of life-threatening bleeding, postoperative atrial fibrillation, and shorter ventilation time, compared to SMVR [9]. TMVR additionally demonstrated superior financial outcomes compared to SMVR as it was linked to shorter pLOS and lower hospitalization costs. The lower level of resource utilization of TMVR may be directly related to the fewer complications experienced by these patients. Additionally, this finding is comparable to previous studies in TMVR as well as transcatheter aortic valve replacement [13, 14, 42]. Compared to surgical aortic valve replacement, transcatheter aortic valve replacement had been demonstrated to be linked to increased costs during the early years. A possible significant contributing factor to the observation was the novelty of the valve and supporting equipment [43, 44]. However, increasing familiarity with the transcatheter approach and continuing advancements in the technology may now contribute to cost mitigation for the transcatheter approach in recent years [42].

Subgroup analysis of transseptal and transapical TMVR approaches revealed TS-TMVR to be associated with decreased odds of mortality. This differs from a prior study by Whisenant et al, who found TS-TMVR to have similar unadjusted mortality rates than TA-TMVR (3.6 vs 6.4%, p = 0.06) [32]. Unlike our study, however, this study did not adjust for factors that may influence mortality. We additionally noted patients undergoing TS-TMVR to have shorter pLOS despite higher adjusted hospitalization costs, compared to their TA-TMVR counterparts [9, 45]. The need for more complex delivery systems and closure of potential iatrogenic atrial septal defects with TS-TMVR may contribute to these cost differences [11]. Although transapical approaches may be required in patients with left atrial thrombus, the results of this study show that TS-TMVR may be the safer, if costlier, approach [45].

Our study has several important limitations inherent to the use of administrative data and its retrospective nature. First, the dataset is based in ICD-10 codes and therefore subject to variation in coding practices. Further, we are unable to account for granular clinical variables including valve-in-valve vs valve-in-ring replacement, transvalvular gradients, ventricular function, and several individual factors. The NRD is similarly limited in description of preoperative risk stratification, as it lacks the variables necessary to calculate the STS Risk Score. Additionally, our results are limited to in-hospital events and readmission during a singular calendar year, limiting comparison of long-term outcomes. Finally, we are unable to determine any causal relationships due to the retrospective nature of the database. Despite these limitations, we utilized robust statistical methodology and the largest national cohort to date to examine contemporary trends and outcomes of TMVR for redo mitral valve operations.

## Conclusions

This study demonstrated that relative to SMVR, TMVR was associated with similar odds of mortality, lower likelihood of complications, and reduced healthcare expenditures. Furthermore, while the transseptal approach is linked to lower adjusted mortality and shorter pLOS compared to transapical TMVR, it remains associated with higher costs. In overall, our findings suggest that TMVR offers a cost-effective and safe treatment method for patients undergoing redo mitral valve operations. Nevertheless, future studies examining long-term outcomes associated with SMVR and TMVR in redo mitral valve operations, are needed.

## Supporting information

S1 Fig Annual trends in the volumes of transapical transcatheter mitral valve replacement (TA-TMVR) and transseptal transcatheter mitral valve replacement (TS-TMVR), 2016–2020. The proportion of TS-TMVR procedures significantly increased over the study period, nptrend < 0.001.
(TIF)

S1 Table ICD10 diagnosis and procedure codes for cohort selection and classification. (DOCX)

S2 Table Demographic, clinical, and hospital characteristics of patients undergoing transapical transcatheter mitral valve replacement (TA-TMVR) or transseptal transcatheter mitral valve replacement (TS-TMVR). (DOCX)

S3 Table Adjusted outcomes of patients undergoing transseptal transcatheter mitral valve replacement (TS-TMVR), compared to transapical transcatheter mitral valve replacement (TA-TMVR). (DOCX)

S4 Table Stata codes for statistical analysis. (DOCX)
