# Supplementary figures and images for: Mortality and resource utilization in surgical versus transcatheter repeat mitral valve replacement: A national analysis
Source: PLoS One. 2024 May 23;19(5):e0301939. doi: 10.1371/journal.pone.0301939 (PMC11115312; doi:10.1371/journal.pone.0301939)

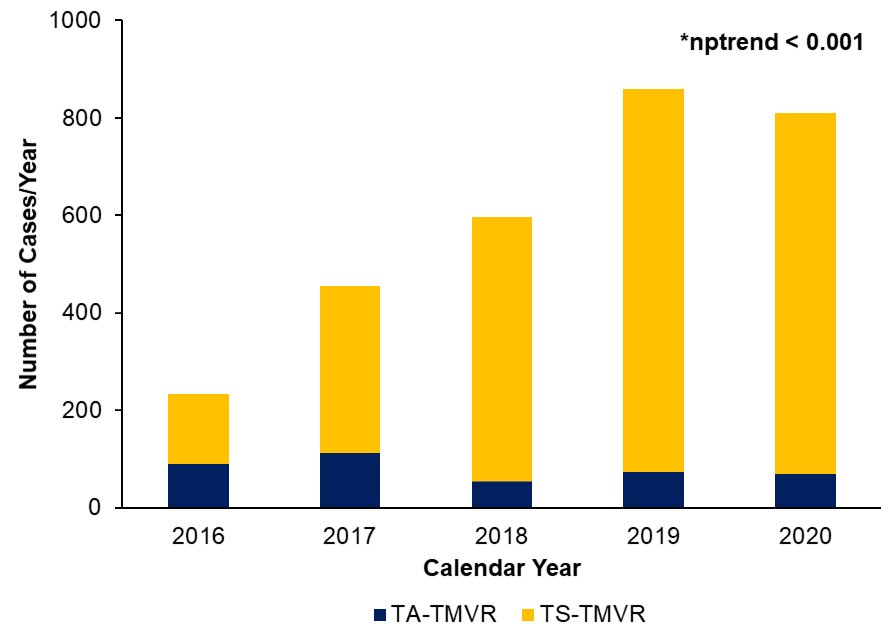

Supplement: S1 Fig — The proportion of TS-TMVR procedures significantly increased over the study period, nptrend < 0.001. (TIF) [file pone.0301939.s001.tif]
