# Supplementary material for: Mortality and resource utilization in surgical versus transcatheter repeat mitral valve replacement: A national analysis
Source: PLoS One. 2024 May 23;19(5):e0301939. doi: 10.1371/journal.pone.0301939 (PMC11115312; doi:10.1371/journal.pone.0301939)
Supplement: S1 Table — (DOCX) [file pone.0301939.s002.docx]

**S1 Table: ICD10 Diagnosis and Procedure Codes for Cohort Selection and Classification**

| **Diagnosis/Procedure Codes** | **ICD-10** |
| --- | --- |
| **Surgical Approaches** | |
| Coronary Artery Bypass Graft | 02100, 02110, 02120, 02130, 02104, 02114, 02124, 02134 |
| Surgical – Aortic Valve Replacement/Repair | 02RF0, 02RF4; 02QF0, 02QF4, 02UF0, 02UF4 |
| Surgical – Mitral Valve Replacement | 02RG0, 02RG4 |
| Surgical – Mitral Valve Repair | 02QG0, 02QG4, 02UG0, 02UG4 |
| Surgical – Tricuspid Valve Replacement/Repair | 02RJ0, 02RJ4; 02QJ0, 02QJ4, 02UJ0, 02UJ4 |
| Surgical – Pulmonary Valve Replacement/Repair | 02RH0, 02RH4; 02QH0, 02QH4, 02UH0, 02UH4 |
| Transcatheter – Aortic Valve Replacement/Repair | 02RF3; 02QF3, 02UF3 |
| Transcatheter – Mitral Valve Replacement | 02RG3 |
| Transcatheter – Mitral Valve Repair | 02QG3, 02UG3 |
| Transcatheter – Tricuspid Valve Replacement/Repair | 02RJ3; 02QJ3, 02UJ3 |
| Transcatheter – Pulmonary Valve Replacement/Repair | 02RH3; 02QH3, 02UH3 |
| **Subgroup of Transcatheter – Mitral Valve Replacement** | |
| Transapical | 02RG37H, 02RG38H, 02RG3JH, 02RG3KH |
| Transseptal | 02RG37Z, 02RG38Z, 02RG3JZ, 02RG3KZ |
| **Diagnoses** |  |
| Bioprosthetic Valve Dysfunction | T8201XA, T8202XA, T8203XA, T8209XA, T82221A, T82222A, T82223A, T82228A, Z952, T82857 |
| Endocarditis | I33, I38, I39 |
| **Comorbidities** | |
| Cardiac Arrhythmia | I441, I442, I443, I456, I459, R000, R001, R008, T821, Z450, Z950, I47, I48, I49 |
| Congestive Heart Failure | I43, I099, I110, I130, I132, I420, I425, I426, I427,  I5022, I5032, I5042 |
| Chronic Lung Disease | I278, I279, J684, J701, J703, J40, J41, J42, J43, J44, J45, J46, J47, J60, J61, J62, J63, J64, J65, J66, J67 |
| Coagulopathy | D65, D66, D67, D68, D691, D693, D694, D695, D696 |
| Diabetes | E100, E101, E109, E110, E111, E119, E120, E121, E129, E130, E131, E139, E140, E141, E149, E102, E103, E104, E105, E106, E107, E108, E112, E113, E114, E115, E116, E117, E118, E122, E123, E124, E125, E126, E127, E128, E132, E133, E134, E135, E136, E137, E138, E142, E143, E144, E145, E146, E147, E148 |
| End-Stage Renal Disease | I120, I131, N250, Z490, Z491, Z492, Z940, Z992, N18, N19 |
| Liver Disease | I864, I982, K711, K713, K714, K715, K717, K760, K762, K763, K764, K765, K766, K767, K768, K769, Z944, K70, K72, K73, K74, B18, I85 |
| Other Neurologic Condition | G254, G255, G312, G318, G319, G931, G934, R470, G10, G11, G12, G13, G20, G21, G22, G32  G35, G36, G37, G40, G41, R56 |
| Pulmonary Circulatory Disease | I26, I27, I280, I288, I289 |
| **Complications** | |
| Stroke/Transient Ischemic Attack | I61, I62, I60, I63, G43601, G43609, G43611, G43619, G973, G9731, G9732, G9751, G9752, G976, G9761, G9762, G9763, G9764, I6781, I6782, I9781, I97810, I97811, G458, G459 |
| Prolonged Ventilation | 5A1955Z |
| Acute Kidney Injury | N17, N170, N171, N172, N178, N179, 5A1D70, 5A1D80, 5A1D90 |
| Reoperation | 0W390ZZ, 0W3B0ZZ, 0W3B3ZZ, 0W394ZZ, 0W3B4ZZ, 02JA0ZZ, 02JA4ZZ, 0W3D0ZZ, 0W3C0ZZ, 0W3C4ZZ, 0W380ZZ, 0W384ZZ, 02JY0ZZ, 02JY4ZZ |
| Vascular Complications | S15, S25, S35, S45, S55, S65, S75, S85, S95, T817, S090, I975, I998, I74, 04Q, 06Q |
| Major Bleeding | K250, K252, K260, K262, K270, K272, K280, K282, K2901, K2921, K2961, K2971, K2981, K2991, K31811, K3182, K5521, K5701, K5711, K5713, K5721, K5731, K5733, K5741, K5751, K5753, K5781, K5791, K5793, K625, K661, K922, I97410, I97411, I97418, I9742, I97610, I97611, I97618, I97620, R040, R042, R049, R58, I6000, I6001, I6002, I6010, I6011, I6012, I6030, I6031, I6032, I604, I6050, I6051, I6052, I606, I607, I608, I609, I610, I611, I612, I613, I614, I615, I616, I618, I619, I6200, I62.01, I6202, I6203, I621, I629, 30230H0, 30230H1, 30230K0, 30230K1, 30230L0, 30230L1, 30230M0, 30230M1, 30230N0, 30230N1, 30230P0, 30230P1, 30230R0, 30230R1, 30230T0, 30230T1, 30233H0, 30233H1, 30233K0, 30233K1, 30233L0, 30233L1, 30233M0, 30233M1, 30233N0, 30233N1, 30233P0, 30233P1, 30233R0, 30233R1, 30233T0, 30233T1, 30240H0, 30240H1, 30240K0, 30240K1, 30240L0, 30240L1, 30240M0, 30240M1, 30240N0, 30240N1, 30240T0, 30240T1, 30243H0, 30243H1, 30243K0, 30243K1, 30243L0, 30243L1, 30243M0, 30243M1, 30243N0, 30243N1, 30243P0, 30243P1, 30243R0, 30243R1, 30243T0, 30243T1 |
