# Supplementary material for: Mortality and resource utilization in surgical versus transcatheter repeat mitral valve replacement: A national analysis
Source: PLoS One. 2024 May 23;19(5):e0301939. doi: 10.1371/journal.pone.0301939 (PMC11115312; doi:10.1371/journal.pone.0301939)
Supplement: S2 Table — (DOCX) [file pone.0301939.s003.docx]

**S2 Table: Demographic, clinical, and hospital characteristics of patients undergoing transapical transcatheter mitral valve replacement (TA-TMVR) or transseptal transcatheter mitral valve replacement (TS-TMVR)**

|  | *TA-TMVR*  (n = 398) | *TS-TMVR*  (n = 2,553) | p-value | |
| --- | --- | --- | --- | --- |
| Age (years, median, IQR) | 75 [69 - 81] | 75 [67 - 81] | 0.38 | |
| Female (%) | 56.3 | 59.9 | 0.33 | |
| Elixhauser Index (median, IQR) | 7 [5 - 8] | 6 [5 - 8] | 0.06 | |
| *Payer Status (%)* |  |  | 0.06 | |
| Private | 8.4 | 9.8 |  | |
| Medicare | 83.9 | 82.8 |  | |
| Medicaid | 3.2 | 5.4 |  | |
| Uninsured/Other | 4.1 | 2.0 |  | |
| *Income Quartile (%)* |  |  | 0.40 | |
| 76th - 100th | 20.8 | 21.1 |  | |
| 51st - 75th | 26.2 | 28.1 |  | |
| 26th - 50th | 31.8 | 26.4 |  | |
| 0 - 25th | 20.9 | 22.8 |  | |
| *Hospital Status (%)* |  |  | 0.03 | |
| Non-Metropolitan | 2.8 | 0.7 |  | |
| Non-Teaching Metropolitan | 8.5 | 6.7 |  | |
| Teaching Metropolitan | 88.7 | 92.6 |  | |
| *Hospital Size (%)* |  |  | 0.002 | |
| Large | 75.4 | 80.1 |  | |
| Medium | 16.2 | 17.2 |  | |
| Small | 8.4 | 2.7 |  | |
| *Comorbidities (%)* |  |  |  |  |
| Cardiac Arrhythmia | 72.8 | 76.9 | 0.23 |  |
| Congestive Heart Failure | 9.2 | 6.3 | 0.18 |  |
| Chronic Lung Disease | 40.1 | 34.0 | 0.11 |  |
| Coagulopathy | 21.7 | 19.1 | 0.42 |  |
| Diabetes | 68.2 | 74.9 | 0.04 |  |
| End Stage Renal Disease | 31.9 | 27.6 | 0.28 |  |
| Liver Disease | 41.8 | 39.7 | 0.61 |  |
| Other Neurologic Condition | 8.0 | 6.8 | 0.53 |  |
| Pulmonary Circulatory Disease | 53.4 | 52.1 | 0.76 |  |

Reported as proportions unless otherwise noted

* IQR, interquartile range
