# Supplementary material for: Mortality and resource utilization in surgical versus transcatheter repeat mitral valve replacement: A national analysis
Source: PLoS One. 2024 May 23;19(5):e0301939. doi: 10.1371/journal.pone.0301939 (PMC11115312; doi:10.1371/journal.pone.0301939)
Supplement: S3 Table — (DOCX) [file pone.0301939.s004.docx]

**S3 Table: Adjusted outcomes of patients undergoing transseptal transcatheter mitral valve replacement (TS-TMVR), compared to transapical transcatheter mitral valve replacement (TA-TMVR)**

|  | AOR/β with 95% CI | p-value |
| --- | --- | --- |
| In-Hospital Mortality (AOR) | 0.44 [0.22, 0.87] | 0.02 |
| *Major Complications (AOR)* |  |  |
| Stroke/TIA | 1.86 [0.39, 8.82] | 0.43 |
| Prolonged Ventilation | 0.54 [0.26, 1.14] | 0.11 |
| Acute Kidney Injury | 0.65 [0.42, 1.00] | 0.05 |
| Reoperation | 1.95 [0.22, 16.92] | 0.55 |
| Major Bleeding | 1.02 [0.57, 1.81] | 0.96 |
| Vascular Complications | 0.87 [0.38, 1.98] | 0.74 |
| *Resource Utilization* |  |  |
| pLOS (β, days) | -0.43 [-0.59, -0.27] | <0.001 |
| Costs (β, $) | 5,200 [300, 10,200] | 0.04 |
| Nonhome Discharge (AOR) | 0.98 [0.53, 1.80] | 0.95 |
| 30-Day Readmission (AOR) | 1.20 [0.79, 1.82] | 0.39 |

Outcomes reported as Adjusted Odds Ratio (AOR) or β Coefficient, with 95% confidence intervals (CI).

* TIA, transient ischemic attack; pLOS, postoperative length of stay
