# Supplementary material for: Mortality and resource utilization in surgical versus transcatheter repeat mitral valve replacement: A national analysis
Source: PLoS One. 2024 May 23;19(5):e0301939. doi: 10.1371/journal.pone.0301939 (PMC11115312; doi:10.1371/journal.pone.0301939)
Supplement: S4 Table — (DOCX) [file pone.0301939.s005.docx]

**S4 Table: Stata codes for statistical analysis**

|  | **Stata Codes** |
| --- | --- |
| **Calculating pLOS** | |
| Determine operative day | gen n_index_day = .  gen placeholder = .  forvalue i=1/15 {  quietly replace placeholder = n_index_day  *Getting index day (Surgical Mitral Valve Replacement)  quietly replace n_index_day=prday`i' if inlist(substr(i10_pr`i',1,5), "02RG0", "02RG4") & (placeholder==. \| prday`i' < placeholder)    *Getting index day (Transcatheter Mitral Valve Replacement)  quietly replace n_index_day=prday`i' if inlist(substr(i10_pr`i',1,5), "02RG3") & (placeholder==. \| prday`i' < placeholder)    } |
| Calculating pLOS | gen postoplos=.  quietly replace postoplos=(los-n_index_day) |
| **Trends** | |
| Trend of TMVR | nptrend mis, by(year) |
| **Cohort Characteristics** | |
| Age | ranksum age, by(mis)  *Median Range  *Open  _pctile age [pweight=discwt] if mis==0, p(25, 50, 75)  return list  *MIS  _pctile age [pweight=discwt] if mis==1, p(25, 50, 75)  return list |
| Elixhauser | ranksum elixsum, by(mis)  *Median Range  *Open  _pctile elixsum [pweight=discwt] if mis==0, p(25, 50, 75)  return list  *MIS  _pctile elixsum [pweight=discwt] if mis==1, p(25, 50, 75)  return list |
| Characteristics | *Characteristics: Female, Income Quartile, Primary Payer, Hospital Teaching Status, Hospital Bed Size  foreach i in female income payer locteach bed {  svy:tab `i' mis, count format(%21x) col missing  } |
| Comorbidities | *Comorbidities: Cardiac Arrhythmia, Liver Disease, Chronic Lung Disease, Coagulopathy, Congestive Heart Failure, Diabetes, End Stage Renal Disease, Other Neurologic Condition, Pulmonary Circulatory Disease  foreach i in CA LD CPD Coagulopathy CHF diabetes RF OND PCD {  svy:tab `i' mis, count format(%21x) col  } |
| **Unadjusted Outcomes** | |
| Clinical Outcomes | *Outcomes: In-hospital Mortality, Stroke/TIA, Prolonged Ventilation, AKI, Reoperation, Vascular Complications, and Major Bleeding  foreach i in died anyneurocomp prolongedven rf_new reoperation VascularComp Bleeding {  svy:tab `i' mis, count format(%21x) col  } |
| pLOS | ranksum postoplos, by(mis)  *Median Ranges  *Open  _pctile postoplos [pweight=discwt] if mis==0, p(25, 50, 75)  return list  *MIS  _pctile postoplos [pweight=discwt] if mis==1, p(25, 50, 75)  return list |
| Cost | ranksum cost_phc, by(mis)  *Open  _pctile cost_phc [pweight=discwt] if mis==0, p(25, 50, 75)  return list  *MIS  _pctile cost_phc [pweight=discwt] if mis==1, p(25, 50, 75)  return list |
| Nonhome Discharge | svy:tab nonhome2 mis, count format(%21x) col |
| 30-Day Readmission | svy:tab readm30ne mis if dmonth<12, count format(%21x) col |
| **Entropy Balancing** | |
| LASSO | *LASSO  splitsample, generate(sample) nsplit (2) rseed(1234)  lasso logit died mis smvr_vol tmvr_vol age female elective income payer locteach bed elixsum CA LD CPD Coagulopathy CHF diabetes RF OND PCD if sample==1, rseed(1234)  estimates store cv  lassoknots, display (nonzero bic)  /*  ---------------------------------------  \| No. of  \| nonzero  ID \| lambda coef. BIC  -------+-------------------------------  2 \| .0368049 1 728.516  6 \| .0253682 2 712.4708  12 \| .0145166 4 702.5822  15 \| .0109813 5 700.2826  20 \| .0068966 6 695.9593  22 \| .0057257 7 700.2993  * 23 \| .005217 7 698.5617  24 \| .0047535 8 704.5322  26 \| .0039465 9 709.5344  27 \| .0035959 12 731.0373  ---------------------------------------  */  lassoselect id=20  estimates store minibic  lassocoef cv minibic, sort (coef, standardized)  lassogof cv minibic, over(sample) postselection |
| Entropy Balancing and Generating New Weights | *Perform entropy balancing  ebalance mis c.smvr_vol c.tmvr_vol c.age female elective c.elixsum LD OND Coagulopathy CPD  *Generating new weights  gen newWeight=discwt*_webal  *Apply new weights  svyset[pweight=newWeight],strata(nrd_stratum) psu(hosp_nrd) singleunit(centered) |
| **Adjusted Outcomes** | |
| Clinical Outcomes | *Outcomes: In-hospital Mortality, Stroke/TIA, Prolonged Ventilation, AKI, Reoperation, Vascular Complications, and Major Bleeding  foreach i in died anyneurocomp prolongedven rf_new reoperation VascularComp Bleeding {  svy:logistic `i' mis, base  } |
| pLOS | svy:poisson postoplos mis, base |
| Cost | svy:regress cost_phc mis, base |
| Nonhome Discharge | svy:tab nonhome2 mis, count format(%21x) col |
| 30-Day Readmission | svy:tab readm30ne mis if dmonth<12, count format(%21x) col |
